# The Design of Poly(lactide-co-glycolide) Nanocarriers for Medical Applications

**DOI:** 10.3389/fbioe.2020.00048

**Published:** 2020-02-11

**Authors:** Divesha Essa, Pierre P. D. Kondiah, Yahya E. Choonara, Viness Pillay

**Affiliations:** Wits Advanced Drug Delivery Platform, Department of Pharmacy and Pharmacology, School of Therapeutic Sciences, Faculty of Health Sciences, University of the Witwatersrand, Johannesburg, South Africa

**Keywords:** poly(lactide-co-glycolide), drug delivery, biodegradable polymer, nanoparticle preparation, nanomedicine, computational simulation

## Abstract

Polymeric biomaterials have found widespread applications in nanomedicine, and poly(lactide-co-glycolide), (PLGA) in particular has been successfully implemented in numerous drug delivery formulations due to its synthetic malleability and biocompatibility. However, the need for preconception in these formulations is increasing, and this can be achieved by selection and elimination of design variables in order for these systems to be tailored for their specific applications. The starting materials and preparation methods have been shown to influence various parameters of PLGA-based nanocarriers and their implementation in drug delivery systems, while the implementation of computational simulations as a component of formulation studies can provide valuable information on their characteristics. This review provides a critical summary of the synthesis and applications of PLGA-based systems in bio-medicine and outlines experimental and computational design considerations of these systems.

## Introduction

The design of novel delivery systems using nanomaterials has experienced substantial growth since the application of nanotechnology to biomedical applications established the field of nanomedicine. As a result of the ongoing discovery of numerous new pharmaceutically active compounds which have shown excellent efficacy but inadequate clinical translation, there is a growing need to fill the gap between the formulations available and their successful inclusion into active treatment. This has urged scientists to investigate alternate forms of delivery to the biological target in order to overcome the hurdles associated with conventional drug delivery, such as poor drug entrapment, inadequate bioavailability and pharmacokinetics, as well as systemic toxicity and side effects. These novel delivery systems all strive for the “magic bullet” effect (Bosch and Rosich, 2008) which is a vehicle that can form favorable interactions with a lipophilic or hydrophilic drug to facilitate high drug loading (Aravind et al., 2013; Bauer et al., 2016), can shield the drug from physiological conditions, deliver it to the biological target with minimal loss, and then can release it at the site in a sustained manner and at therapeutic concentrations (Al-Jamal et al., 2016; Almoustafa et al., 2017). Moreover, the carrier is ideally biodegradable, biocompatible and non-immunogenic, with low systemic toxicity (Alshamsan, 2014; Ananta et al., 2016). Nanomaterials are a befitting source to meet these requirements because they can be tailored to a vast range of sizes and shapes and can suit various delivery mechanisms, while the interactions between the carrier and the physiological medium can be controlled by adapting the surface properties of the carrier (Kakkar et al., 2017). This has given rise to the widespread implementation of nanomaterials as pharmaceutical carriers for medical diagnostics and therapeutics (theranostics) (Berthet et al., 2017; Singh et al., 2019). Nanostructures can be fabricated from organic, inorganic, metallic or non-metallic sources. Examples include carbon nanotubes (Singh et al., 2013), dendrimers, liposomes, micelles, and solid lipid nanoparticles (Mishra et al., 2014; Lombardo et al., 2019).

Polymeric nanoparticles are commonly implemented as components of drug delivery systems and the use of synthetic polymers in particular can enable the design of carriers in a well-controlled and reproducible manner in order to suit the desired application (Lai et al., 2014). Polymer-based nanoparticles act as drug delivery vehicles by encapsulating the active agent inside its polymeric matrix, by conjugating to the agent or by adsorbing it onto the surface of the polymer (Mahapatro and Singh, 2011; Ansary et al., 2014). Polymers can be constructed to be linear, branched or globular and their size and their properties can be modulated by the choice of synthetic process (Panyam and Labhasetwar, 2012; Chen et al., 2013). Biodegradable polymers are suitable as nanocarriers as they often self-assemble and are easily sourced. Natural biodegradable polymers are chitosan (Ali and Ahmed, 2018), alginate (Jana et al., 2016) and inorganic ceramic hydroxyapatite composites (Turon et al., 2017). Synthetic polymers such as poly lactide-co-glycolide (PLGA) are an attractive alternative as they can be precisely engineered from monomers to suit the target and physiological environment they are intended for Middleton and Tipton (2000).

**FIGURE 1:**
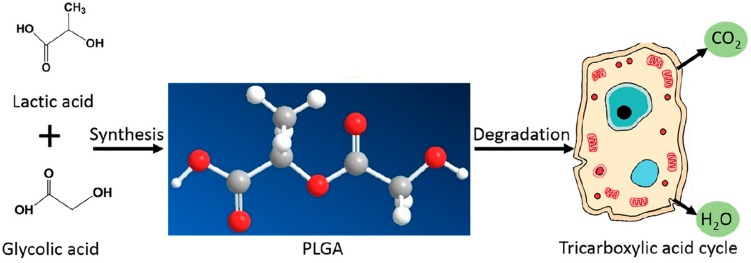
The synthesis and physiological breakdown of PLGA, adapted from Sun et al. (2017).

Poly lactide-co-glycolide has become ubiquitous in the bio-medical field for many reasons. Firstly, it is a synthetic, biodegradable polymer that is easily broken down *in vivo* by hydrolysis into lactic acid and glycolic acid. These monomers are biocompatible and are physiologically metabolized by the tricarboxylic acid cycle for final excretion in the lungs (Semete et al., 2010; Sequeira et al., 2018), as shown by Figure 1 (Sun et al., 2017). Hence, PLGA as a nanocarrier is considered to produce minimal systemic toxicity when used for biomedical applications (Kumari et al., 2010) and has been used in various formulations including membranes, sponges and gels (Sun et al., 2017).

The appeal of PLGA also lies in the fact that its properties can be manipulated and adapted to modify the encapsulation profile and drug release kinetics of the nanostructure to suit the desired application (Mittal et al., 2007). PLGA is overall a hydrophobic polymer and is therefore detected by the RES and if unmodified, is bound by phagocytes for elimination by the liver or spleen and eliminated before delivering its payload to the target site (Danhier et al., 2012). To circumvent this, surface modification of PLGA is necessary. One such modification is the coating of hydrophilic poly ethylene glycol (PEG) groups on the surface of PLGA to shield the hydrophobic end groups from the reticulo endothelial system (RES), resulting in an amphiphilic di-block co-polymer (Salmaso and Caliceti, 2013). Other polymers used as surface modifiers include chitosan (Lu et al., 2019), polaxamer and poloxamines (Redhead et al., 2001) which work by altering the electrostatic and hydrophobic surface properties of the PLGA block co-polymer. To increase the therapeutic efficacy, the surface of the PLGA nanocarrier can be decorated with targeting ligands such as small molecules, antibodies, and aptamers. These molecules selectively bind to receptors on the target cell and guide the vehicle to the site of action (Jahan et al., 2017). Targeting moieties such as aptamers, have been shown to increase retention time at the site of action (Dinarvand et al., 2011).

**FIGURE 2:**
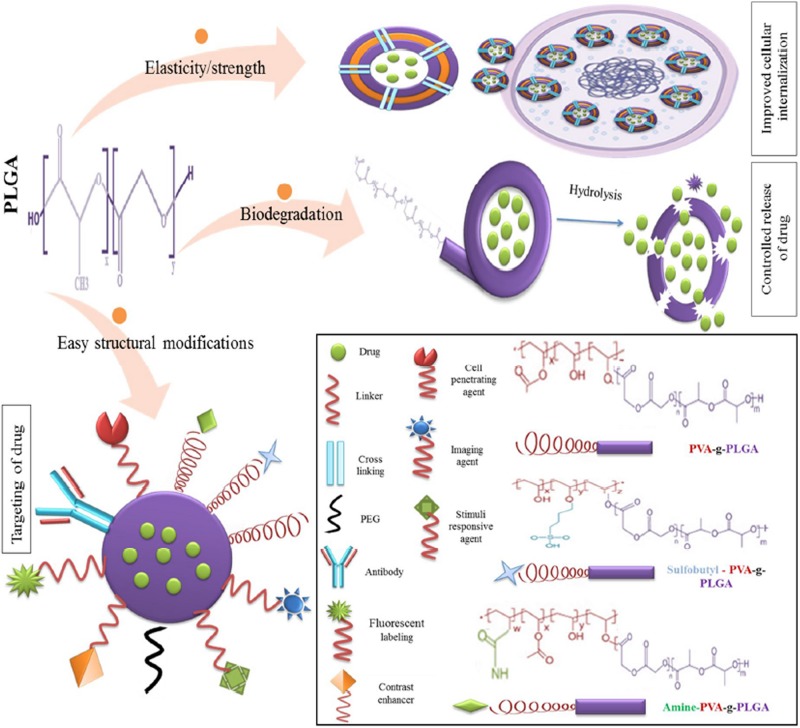
Favorable characteristics of PLGA as a nano delivery system, reproduced from Mir et al. (2017).

The use of PLGA in biomedicine dates back to the 1970s when it was used as a component of biodegradable sutures and implants. With the advent of nanomedicine, it has found application as nanocarrier in various areas of medical research, including chemotherapeutics, immunology, and biomechanics (Swider et al., 2018). Numerous studies have also reported successful applications in antibiotics, antiseptics, imaging, wound healing, and as nano scaffolds (Sharma et al., 2016). The suitability and adaptability of PLGA as a nanocarrier is illustrated by Figure 2 (Mir et al., 2017).

Optimizing the synthetic procedure by changing the parameters can affect other properties of nanocarriers and therefore a great deal of forethought should go into the design of the system for the particular application (Rezvantalab et al., 2018). During synthesis, parameters such as particle size, surface behavior, degree of crystallinity, degradation rate, and molecular weight can be modified to adapt the nanocarrier for desired dosage and site specific action (Mittal et al., 2007). Bio-nano interactions are important considerations in design as they determine the suitability of the nanostructure for the intended application as well as the undesired toxicity that may result from the engineering process. Previous research has indicated accumulation of PLGA in the liver when used as nanocarriers and therefore there could be toxicity challenges caused by dose dumping (Makadia and Siegel, 2011). While there are numerous reviews on PLGA based nanodelivery systems in general, this work considers the literature from a design perspective. Schrur’s nano-toxicology editorial states “few studies offer consistent results that are of value, and it is difficult to compare studies because they are often carried out using poorly characterized nanomaterials and arbitrary experimental conditions” (Schrurs and Lison, 2012). With these considerations in mind, *in silico* design, which is an expanding field in drug delivery, could be used to model numerous parameters, including polymer degradation, drug loading and toxicity and hereby provide insight into the structure-behavior relationships of PLGA-based nanocarriers (Ramezanpour et al., 2016). The aim of this review is to collate research on PLGA based delivery vehicles that have been studied for common medical applications, to compare the choices of starting materials and synthetic methods on the properties and functions of the final polymer-drug systems, and to explore how computational investigations can assist in the design of these systems.

## PLGA as a Nanocarrier

### Properties

Poly lactide-co-glycolide is synthesized from its constituent monomers (Sun et al., 2017) and can be obtained commercially in varying ratios of these monomers (Sadat Tabatabaei Mirakabad et al., 2014). Each constituent has its own physical characteristics that it brings to the co-polymer. PLGA retains properties of both copolymers and can be customized using these properties, which are stiff, hydrophobic and slowly degrading lactic acid vs. malleable, less hydrophobic and faster degrading glycolic acid (Engineer et al., 2011). For example, poly-DL -lactic acid has a methyl group, as shown in Figure 1, and is therefore more hydrophobic than poly glycolic acid. Hence, adjusting the concentration of poly-lactic acid in PLGA varies the solubility of the final polymer (Makadia and Siegel, 2011). A study investigating the rate of hydrolysis of PLGA demonstrated that increasing glycolic acid to lactic acid ratio increases the hydrophilicity of the PLGA co-polymer and hence leads to faster degradation (Keles et al., 2015), while a separate study quantified the degradation constant to be 1.3 times higher for glycolic units than for lactic units in the PLGA co-polymers investigated (Vey et al., 2011). It has been shown that PLGA co polymer ratios can be varied to adapt the degradation rate from months to years (Sun et al., 2017). In general, the higher the glycolic acid content of the PLGA polymer, the more amorphous it is and the faster it degrades due to it being more hydrophilic. An exception is PLGA 50:50 lactic: glycolic units, which has exhibited the fastest degradation rate (Lü et al., 2009). It has been shown that increasing the glycolic acid ratio increases the wettability of PLGA for thin film applications (Ayyoob and Kim, 2018) and that increasing the lactic acid content has application in designing PLGA carriers for sustained release (Li, 1999). PLGA co-polymers with lactic acid content less than 70% have been characterized as amorphous and suitable for drug delivery applications (Habraken et al., 2006). As expected, the higher the molecular weight of PLGA, the more structural integrity it exhibits and the longer it has shown to degrade *in vivo* (Anderson and Shive, 2012). The PLGA co-polymer can be end-capped with different functional groups which have shown to affect the degradation kinetics of the delivery system. For example ester end-capped polymers exhibit a slower degradation rate than acid end-capped polymers and are therefore suitable for slower release applications (Gentile et al., 2014). Apart from degradation rate, it is also possible to control solubility and glass transition temperature of the PLGA system by varying the molecular weight, lactic/glycolic ratios, and end-cap functional groups of the starting material (Gentile et al., 2014).

Poly lactide-co-glycolide is also soluble in a variety of organic solvents including acetone, dichloromethane, chloroform, ethyl acetate, and THF (Sharma et al., 2016) and therefore is relatively simple to work with as carriers for both hydrophobic and hydrophilic drugs (Zhang et al., 2014).

### Surface Functionalization

#### Shielding

In order to avoid elimination by the RES, a stealth coating around the hydrophobic PLGA nanoparticle surface has been achieved by incorporation of co-polymers with desired properties. The most frequently used co-polymer is polyethylene glycol (PEG) as it is biocompatible and easily grafted or adsorbed onto the surface of PLGA. The hydrophilic PEG shields the PLGA carrier from being taken up by opsonins (Vllasaliu et al., 2014) and it has been shown that the PEG shield dramatically increases the blood circulation half-life of the nanocarrier (Owens and Peppas, 2006). Some studies have shown that the nanoparticle *in vivo* residence time is dependent on the surface density of the PEG chains (Bertrand et al., 2017). PEGylation has also been associated with enhanced drug loading and tunable carrier degradation (Khalil et al., 2013). Chitosan, a natural polymer that is formed by partial deacetylation of chitin, is also commonly grafted onto the surface of PLGA based systems to increase biocompatibility. It is biodegradable and has mucoadhesive properties as it carries a positive charge and can efficiently bind to negatively charged cell membranes (Bruinsmann et al., 2019). Hence, a coating of chitosan on the PLGA nanostructure shields it from opsonins and promotes stronger cellular interaction and retention (Lima et al., 2018). Collagen is a highly hydrophilic protein that also increases cellular interaction and when blended with PLGA, can form a delivery system with superior hybrid properties such as increased biological compatibility and mechanical strength (Sadeghi-Avalshahr et al., 2017). Heparin, a biocompatible material that can be obtained both naturally and synthetically, has been used to impart specific binding properties to delivery systems when combined with polymers (Rodriguez-Torres et al., 2018). It is a sulfated glycosaminoglycan with high binding affinity for various growth factors and has been used in sustained delivery applications by immobilization on the surface of PLGA delivery systems (Chung et al., 2006).

#### Surfactants

One of the strategies employed in the nanofabrication process to increase colloidal stability is the use of surfactants. These are agents which usually have amphiphilic properties and reduce the interfacial tension between the hydrophobic and hydrophilic components, hence increasing miscibility and dispersion (Heinz et al., 2017) and preventing particle aggregation (Shkodra-Pula et al., 2019). A commonly used agent is polyvinyl alcohol (PVA), which is a hydrophilic polymeric surfactant that has been shown to decrease the size and increase the uniformity of PLGA nanocarriers, but is also associated with hypertension and central nervous system depression in animal studies (Menon et al., 2012). The use of Polysorbate 80, 60, and 20 has also shown increased residence time and enhanced permeation of the blood brain barrier (Sharma et al., 2016). However, it has shown in some cases to cause anaphylactoid reactions (Coors et al., 2005) and long-term infertility (Gajdova et al., 1993). Polaxamer is a thermo-reversible, non-toxic coating that has been used when encapsulating hydrophobic drugs and has been shown to preferentially target cancer cells. However, it has shown rapid erosion times and it is associated with hyperlipidemia and hypercholesterolemia (Miller and Drabik, 1984). Poloxamine is an amphiphilic block co-polymer and therefore has been used to stabilize hydrophobic drugs while increasing circulatory residence time (Alvarez-Lorenzo et al., 2010). Vitamin E TGPS is a water-soluble form of vitamin E and is used as a solubilizing and emulsifying agent in nano drug delivery. It is commonly used to enhance drug loading (Zhang and Feng, 2006) and nanoparticle degradation rates (Jalali et al., 2011).

#### Active Targeting

**FIGURE 3:**
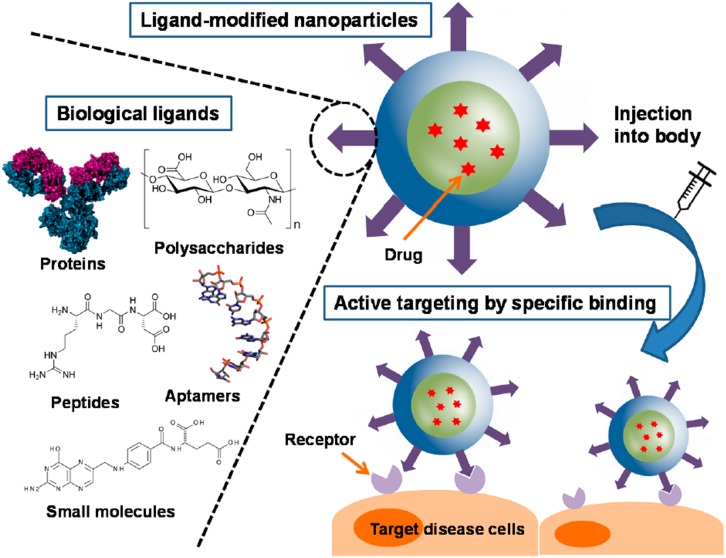
Surface functionalized PLGA nanoparticles for active targeting and cellular binding reproduced from Yoo et al. (2019).

In active targeting, the surface of the nanoparticle is further decorated with ligands that specifically bind to receptors on the cells of interest and enables the carrier to enter the cell by receptor mediated endocytosis (Muhamad et al., 2018). These targeted delivery systems are designed to localize drug release at the disease site (Danhier et al., 2010). There are various different kinds of targeting ligands such as small molecules, peptides, antibodies, aptamers and polysaccharides, as shown in Figure 3 (Yoo et al., 2019). These ligands can be either conjugated or adsorbed onto the surface of the nanocarrier after formation or can be linked to one of the components of the carrier before nanoparticle formation (Yoo et al., 2019). It has been shown that increasing the conjugation density of targeting ligands has an effect on the targeting ability of the nanocarrier.

Monoclonal antibodies have had a long history as targeting ligands (Friedman et al., 2013) since they have complementarity determining regions that enable them to bind to receptors on cell surfaces with high specificity and affinity (Carter et al., 2016). However, since they are large molecules, their conjugation density capacity on the nanocarrier is substantially decreased (Yoo et al., 2019) compared to other ligands, and furthermore, they raise immunogenicity concerns (Karra and Benita, 2012). Compared to antibodies, peptides have the advantage of smaller size and non-immunogenicity but they still are able to retain target specificity (Zhao et al., 2007). Aptamers are short strands of nucleic acids that can be synthetically designed to bind specific biological targets. They are non-immunogenic and non-toxic but their synthesis can be costly (Friedman et al., 2013). Despite their advantages for *in vivo* targeting, both peptides and aptamers are prone to enzymatic degradation (Yoo et al., 2019). Polysaccharides are advantageous because they are biocompatible and can be used as structural components of the nanocarrier (Choi et al., 2011) as well as to target carbohydrate binding receptors on cell surfaces. However, some polysaccharides could have solubility challenges and modification of the carbohydrate structure could result in unintended toxicity (Peng et al., 2018). Small molecules form a class of targeting ligands that comprise of synthetic compounds that are designed to target certain domains on cell surface receptors. They are usually chosen because of ease and control of synthesis but they often do not bind with high specificity and some target receptors can be expressed in healthy cells (Yoo et al., 2019), resulting in unintentional cell binding. Even though active targeting strategies provide an attractive avenue for site specific drug delivery, there are many challenges in this area, such as receptor accessibility and off-target binding. Particularly during different stages of tumor development, certain receptors can be up or down regulated, which provides an additional challenge for the use of targeting ligands in chemotherapeutic drug delivery (Vhora et al., 2014).

### Toxicity

Despite biocompatibility and biodegradability of PLGA as a polymer, its toxicological profile in nanoformulations deserves to be investigated because of altered physicochemical properties, such as higher surface area to mass ratios. Furthermore, reports have suggested that particles of any material may acquire unique toxicological properties in the nanoscale (Makadia and Siegel, 2011). Different effects such as acute toxicity, repeated dose toxicity, inflammation, oxidative stress, genotoxicity, and reproductive system toxicity of PLGA nanocarriers have been examined in order to obtain information on the possible risks of these materials in pharmaceutical preparations. A study of danorubicin loaded PEG-PLL-PLGA nanoparticles has described some toxicity in Kunming mice (Guo et al., 2015) but since no results were reported for blank nanoparticles, it is unclear whether the toxicity was due to the drug or nanocarrier (Jesus et al., 2019). Regarding oxidative stress, studies have reported an overall increase in the production of reactive oxidative species corresponding to increasing concentrations of the PLGA nanoformulations tested (Singh and Ramarao, 2013; Grabowski et al., 2015) and another study demonstrated mild inflammatory properties of different PLGA formulations (Grabowski et al., 2016). Several studies have confirmed no genotoxicity (Tulinska et al., 2015; Platel et al., 2016), no toxicity on reproduction (Chen et al., 2017; Sharma et al., 2017) and no hemolysis (Chen et al., 2017). A study comparing the toxicity of PLGA nanoparticles to silica-, iron-, and zinc-based nanoparticles showed that the PLGA system had no appreciable adverse *in vitro* or *in vivo* toxicological outcomes, and did not produce the toxicity commonly associated with the inorganic nanomaterials (Semete et al., 2010).

## Synthetic Methods of PLGA Nanocarriers

**FIGURE 4:**
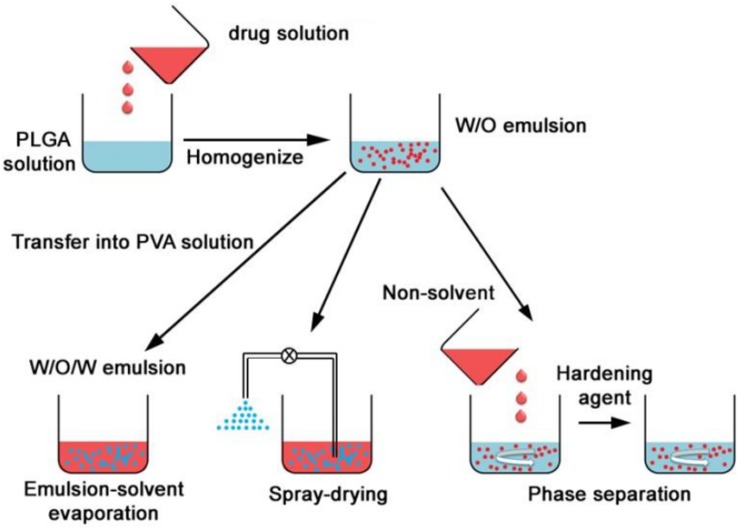
Formation of PLGA nanoparticles by double (W/O/W) emulsion, spray drying and phase separation (coacervation) methods, adapted from Ding and Zhu (2018).

Poly lactide-co-glycolide nanocarriers may be fabricated by different methods and the choice of method has shown to affect properties such as particle size, colloidal stability, drug loading/encapsulation efficiency, and release behavior of the final product (Swider et al., 2018). Depending on the process of preparation, the structural organization may also be different. The drug is either encapsulated inside the carrier or adsorbed on the surface (Danhier et al., 2012). There are several methods that can be employed for the preparation of PLGA nanocarriers, and the following provides a brief overview on both the well-established and relatively recently developed techniques. The traditional methods based on emulsions are illustrated by Figure 4 (Ding and Zhu, 2018).

### Single and Double Emulsion

The emulsion methods have been the most frequently used methods of synthesis and they are suitable for a wide range of drugs with varying solubilities (Wang et al., 2016). The single emulsion (oil in water or O/W) method is suitable for hydrophobic drugs. PLGA and the drug is dissolved in a small volume of suitable volatile organic solvent and added dropwise to the aqueous phase containing a stabilizer, usually PVA. The mixture is sometimes sonicated and then stirred, often under sheer stress for a fixed amount of time to allow the organic solvent to evaporate. The double emulsion method is used when the active agent to be entrapped is hydrophilic, such as proteins and peptides. The active is dissolved in an aqueous phase and then added to PLGA which is dissolved in the organic phase, and this forms a primary water in oil (W/O) emulsion. This is then added to another aqueous phase containing a stabilizer and allowed to mix under stress, allowing the organic solvent to evaporate. The nanoparticles are therefore formed by a water in oil in water (W/O/W) emulsion (Makadia and Siegel, 2011). The product is isolated by centrifugation or ultrafiltration and washed to remove unreacted products. Thereafter it is freeze dried and can be stable for several months to years. Recently, a single emulsion method was used to successfully entrap proteins for vaccine application (Ospina-Villa et al., 2019) and a PLGA-PEG nanocarrier was formulated using the double emulsion method for intraperitoneal insulin delivery (Haggag et al., 2018). The emulsion methods can be adjusted by changing the drug to PLGA ratio, the organic solvent, the stabilizer concentration in the aqueous phase and the stirring speed and can hereby be adapted to control the size range of the nanocarriers to some extent. However, there are often batch to batch variation with these methods and the carriers prepared by this method for protein-based drugs have limited stability due to degradation of proteins at the aqueous interface and the sheer stress of homogenization leading to unfolding of the protein sheets (Ding and Zhu, 2018).

### Spray Drying

This method involves the preparation of water in oil or solid in oil emulsions, which are sprayed in a thin stream of heated air. The type of drug (hydrophilic or hydrophobic) would determine the solvent used in the emulsion (Makadia and Siegel, 2011). Recently, spray drying was used in the preparation of a PLGA nanoformulation for sustained treatment of tuberculosis (TB) (Kalombo et al., 2019) and in the fabrication of a carrier for antibiotic coating of dental implants (Baghdan et al., 2019). This method is highly advantageous because it is suitable for hydrophobic and hydrophilic drugs and can be used for sensitive compounds since the conditions are mild. It is also a rapid method (Nie et al., 2008) which can be suitable for industrial scale-up due to the minimal processing parameters involved (Ding and Zhu, 2018). The main drawback of this technique is the wastage caused by inaccessible product that adheres to the inside of the nanosprayer (Wang et al., 2016). Parameters such as orientation of jets, temperature, and solvent choice can all affect the properties of the final nanoparticles (Berkland et al., 2004).

### Coacervation

With coacervation or phase separation, the polymer and drug are prepared as O/W for hydrophobic drugs and W/O/W for hydrophilic drugs, and then a non-solvent, e.g., silicon oil is added dropwise under stirring (Verma et al., 2018). This reduces the solubility of PLGA in the organic solvent and results in the formation of a polymer-rich phase in which PLGA surrounds the drug molecules to form microdroplets (coacervates). These are rapidly quenched in a non-soluble medium to form the solid product (Wang et al., 2016). Parameters such as starting polymer, solvent choice, stirring rate and temperature can be varied to control the properties of the particles. Coacervation usually forms micrometer sized particles (Sharma et al., 2016) but has been used in protein nanoparticle preparation (Verma et al., 2018).

### Salting Out

**FIGURE 5:**
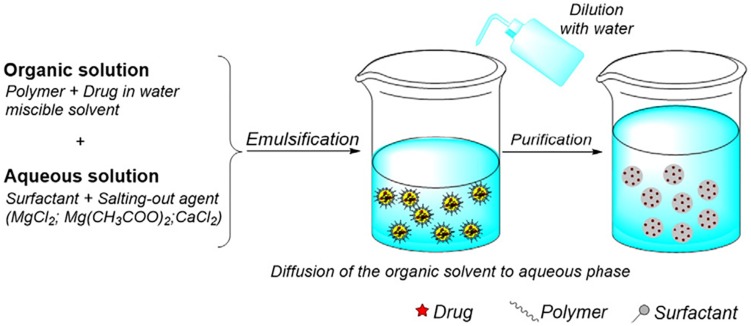
Illustration of the salting out method of preparation, reproduced from Crucho and Barros (2017).

In the salting out method, drug and PLGA are dissolved in a miscible organic solvent and added to the aqueous phase containing stabilizer and a salt under sheer mechanical force. The salt usually used is magnesium chloride hexahydrate or magnesium acetate tetrahydrate (Wang et al., 2016) and is used at a ratio of 1:3 PLGA:salt (Eley et al., 2004). Upon addition of water, the organic solvent diffuses into the aqueous phase, causing the formation of PLGA-drug nanoparticles, illustrated by Figure 5 (Crucho and Barros, 2017). This method is not suitable for lipophilic drugs and can be time intensive since isolation of the product involves several washing steps to remove reagents. However, it would suit drugs which are very temperature sensitive since heat is not required (Nagavarma et al., 2012). This method is robust and is suitable for nanoparticles with high polymer concentrations since the size of the particle is not generally affected by the amount of polymer (Swider et al., 2018).

### Nanoprecipitation

**FIGURE 6:**
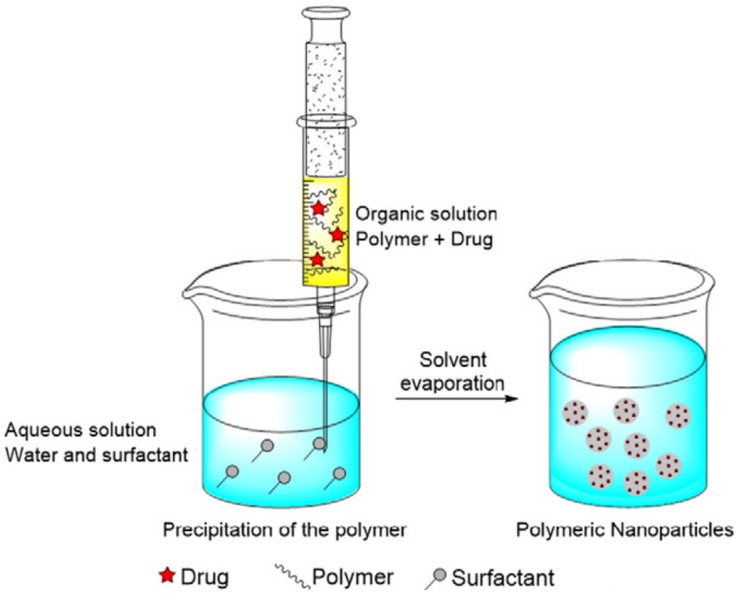
The nanoprecipitation method, adapted from Crucho and Barros (2017).

In this method, PLGA and the drug is dissolved in a polar, water miscible solvent and added dropwise to the aqueous phase, which may contain a surfactant. The product is formed by rapid diffusion of the water miscible solvent into the aqueous phase, resulting in precipitation of the PLGA-drug nanoparticles, as shown in Figure 6 (Crucho and Barros, 2017). The properties of the nanocarrier are controlled by PLGA content and molecular weight, PLGA to drug ratio and choice of solvent (Wang et al., 2016). Recently, an optimized nanoprecipitation method was developed for the preparation of PLGA encapsulated alendronate sodium, a drug for osteoporosis (Oz et al., 2019) and a modified procedure was reported for a PLGA hybrid nanocarrier for simvastatin (Zhang et al., 2018). Nanoprecipitation can be used to prepare particles in the 100 nm size range and is advantageous because of the absence of shear stress (Fessi et al., 1989). However, the unmodified nanoprecipitation method does not usually work well for hydrophilic drugs as they do not form favorable interactions with PLGA in a water miscible solvent (Govender et al., 1999).

### Supercritical Fluid Technology

**FIGURE 7:**
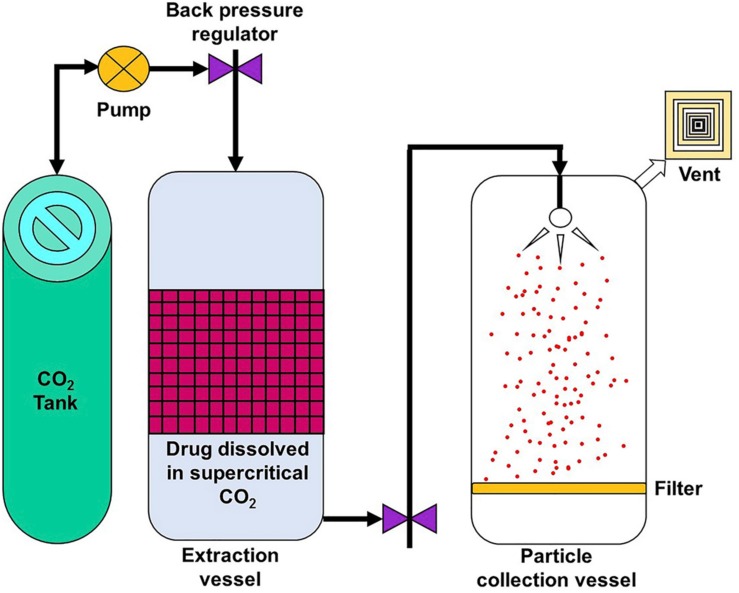
Simple setup of a supercritical fluid technology method, reproduced from Jog and Burgess (2017).

Supercritical fluid technology, illustrated by Figure 7 (Jog and Burgess, 2017), can provide an environmentally friendly method of generating nanoparticles since it reduces, and in some cases, eliminates the use of organic solvents (Koushik and Kompella, 2004). In this method, the polymer and drug are dissolved in a supercritical fluid which is then rapidly expanded and depressurized. The resultant mixture is then passed through a fine nozzle or capillary, resulting in supersaturation and formation of nanoparticles, which are collected separately (Mishima, 2008). This is an attractive method as it is highly tunable but the kind of nanoparticle products are restricted since not all starting materials are compatible with the supercritical fluid (Soh and Lee, 2019).

### Microfluidics

**FIGURE 8:**
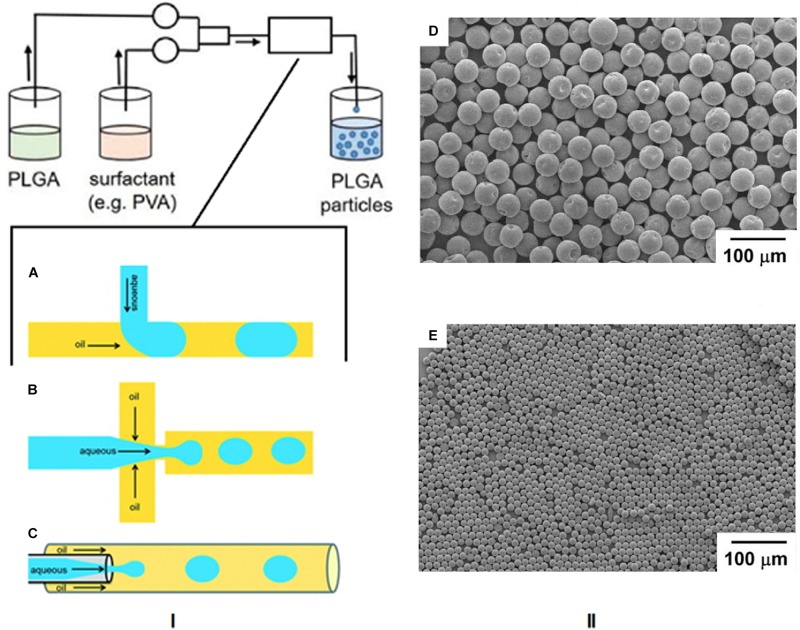
**(I)** Schematic of the microfluidics method and the three most common microchannel geometries. **(A)** T junction, **(B)** flow focusing, **(C)** continuous flow. Reproduced from Shembekar et al. (2016)-published by the Royal Society of Chemistry. **(II-D,E)** SEM images of PLGA particles formed by this technique, adapted from Xu et al. (2009).

The area of microfluidics deals with channels of the micrometer size range that are used to control and manipulate the movement of volumes of fluid from the nanolitre size range and below. When working at the nanoscale, the conditions of flow can be precisely controlled and constant laminar flow is maintained, which is impossible when conducting reactions at the macroscale level (Chiesa et al., 2018). Therefore this technique has lent itself to the synthesis of nanoparticles by the formation of emulsions using droplet microfluidics. In this method, the polymer and drug are combined and the emulsion is formed in the microfluidic mixer, which can have different channel architechtures (Collins et al., 2015). The most commonly used geometries for droplet based PLGA nanoparticle formation are the t-junction, flow focusing, and continuous flow microchannels, as shown in Figure 8I (Shembekar et al., 2016). In the t-junction geometry, channels are perpendicular to each other. The dispersed phase (aqueous) flows through one channel while the continuous phase (oil) flows through the other and droplets are formed at the junction. In the flow focusing system, the aqueous phase flows through a square capillary where shear force is provided on either side of it by the flow of the oil phase. The emulsion then flows through a narrow capillary and droplets are formed in a collection chamber. With continuous flow geometry channels, the aqueous phase flows through a capillary that resides in another capillary through which the oil flows in the same direction. Droplets begin to form once the two phases mix (Shembekar et al., 2016). SEM images of particles formed by this method are shown in Figures 8II-D,E (Xu et al., 2009).

These microchannels are used for O/W emulsions, but can be adapted for double (W/O/W) emulsions by using a combination of channels. Recently, a microfluidics method was developed for the encapsulation of cell penetrating peptides (Streck et al., 2019) and targeted delivery of taxanes (Martins and Sarmento, 2020). There are numerous advantages of microfluidics for nanoparticle synthesis. With this technique, the chemical composition of the final product can be preselected according to the desired application. The synthetic parameters can be controlled to the extent that there is a much larger particle size homogeneity compared to bulk methods, and there are also smaller volumes of solvent needed. However, the scale of nanoparticle production is limited and the microchannels are also susceptible to blockage and contamination. The time and temperature of mixing, flow rate, choice of solvents and payload type determine the properties of the final nanoparticles (Kim K.T. et al., 2019).

### Membrane Extrusion Emulsification

**FIGURE 9:**
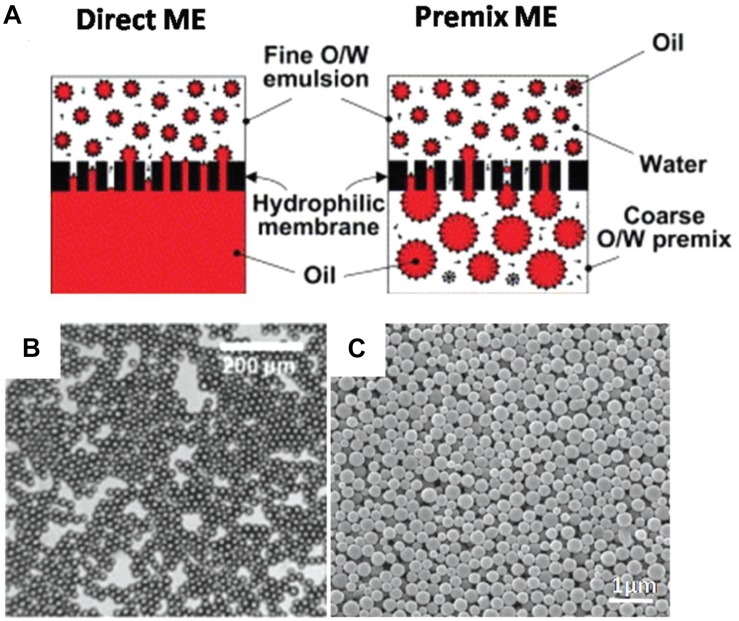
**(A)** Direct- and premix- two common processes using membrane extrusion (ME). **(B)** SEM image of an emulsion formed by this method **(C)** SEM images of nanoparticles formed by premix ME, reproduced from Guo et al. (2018).

With this technique, single or double emulsions of PLGA and drug are either initially prepared or formed when extruded through a membrane of predetermined pore size. There are two ways to do this – direct and pre-mix membrane extrusion (ME), as illustrated by Figure 9A (Guo et al., 2018), and a characteristic emulsion is shown in Figure 9B. In direct ME, the membrane emulsifies the dispersed phase into nanosized droplets, while in premix ME, the emulsion is formed via a conventional method and thereafter extruded through the membrane, which downsizes the coarse emulsion into uniform nanosized droplets. This method can be used for hydrophobic or hydrophilic drugs and is advantageous because the size of product can be controlled by varying the nanoporous membrane pore size to create particles of required dimensions, resulting in a large size homogeneity. Premix ME in particular has been shown to have a higher uniformity in dispersity of final nanoparticles as shown by Figure 9C, compared to direct ME. In general, it is a mild procedure with low energy requirements and can be easily scaled up. However, it is not suitable for emulsions with high viscosity (Guo et al., 2018).

### Nanoimprint Lithography and the PRINT Technique

**FIGURE 10:**
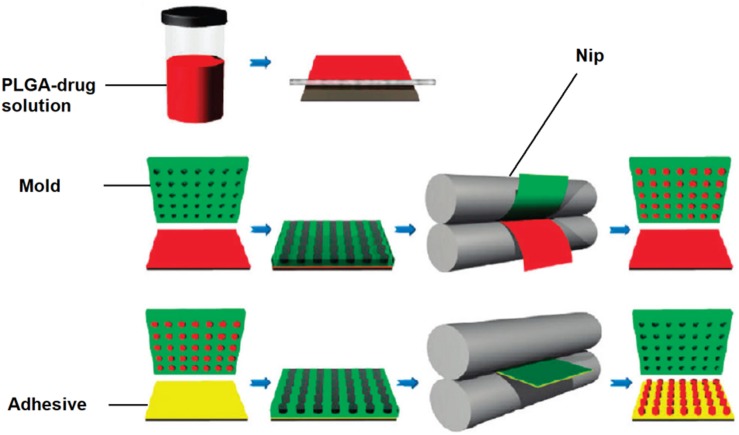
Schematic showing PRINT procedure, reprinted with permission from Perry et al. (2011). Copyright (2019). American Chemical Society.

**FIGURE 11:**
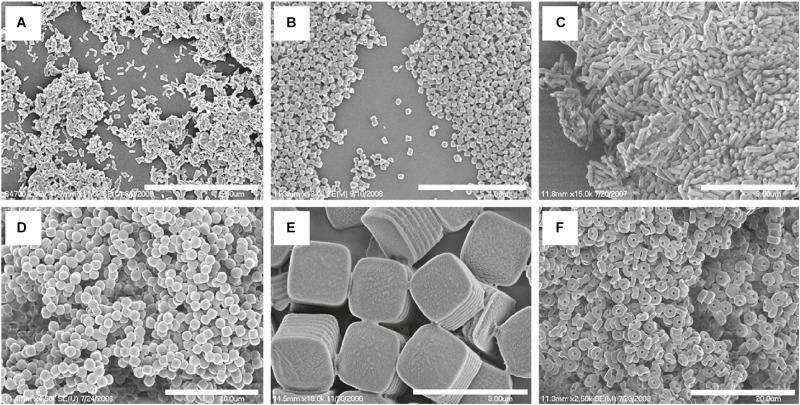
SEM Images of PLGA PRINT particles. **(A–C)** Cylinders of different dimensions. **(D)** Spheres; **(E)** ridged cubes; (**F**, bottom right) particles with center fenestrations, reprinted with permission from Enlow et al. (2011). Copyright (2019) American Chemical Society.

Nanoimprint lithography is used to form nanoparticles from a nanostructure template that is placed over a layer of precursor material which is heated to above the glass transition temperature of the polymer. Thereby, the malleable precursor material is molded into the desired size and shape, which is retained upon cooling. The template is then removed, leaving the product on the substrate base. Su et al. (2015) have successfully used this method for the nanofabrication of submicron PLGA grooves for the control of the length and direction of retraction fibers during cell division. The major drawback of this method is the residual interconnecting layer on the substrate base that prevents the formation of isolated nanostructures (Fu et al., 2018). The PRINT (particle replication in non-wetting template) technique involves the preparation of the PLGA-drug solution matrix and casting it on a delivering sheet. Thereafter a mold with nanosized cavities is placed over the delivering tray and it is passed through a nip and separated so that the polymeric material fills the mold cavities. The particles are then solidified and placed on a high energy adhesive layer and passed through the nip without separation. After the mold is removed, the nanoparticles are collected by washing with a solvent that dissolves the adhesive (Perry et al., 2011). The method is automated with a high degree of control over the individual parameters, and can be used for a wide variety of cargos including hydrophobic and hydrophilic drugs, vaccines, and proteins. However, it is a multistep process that can be labor intensive (Swider et al., 2018). The desired particle size, surface properties and composition can be preset and controlled in the initial step. The PRINT process is illustrated in Figure 10 (Perry et al., 2011), while Figure 11 shows SEM micrographs of the different shapes of PLGA nanoparticles that have been prepared by this method. Enlow and colleagues have reported the PRINT process whereby PLGA micro- and nanoparticles were prepared, with cylindrical, spherical, ridged, and fenestrated morphologies. These particles demonstrated > 40% drug loading and > 90% encapsulation efficiencies of docetaxel (Enlow et al., 2011).

## Medical Applications

### Cancer Research

#### Actively Targeted Chemotherapeutics

In the United States of America alone, 1,762,450 new cancer diagnoses and 606,880 cancer related deaths are expected to occur in 2019 Siegel et al. (2019). Despite the ubiquity of this disease, treatment options are challenging due to the complex pathology of the different cancers. Current chemotherapy often leaves debilitating and life altering side effects since most drugs on the market that target the rapidly dividing cancer cells also inadvertently damage cells that are vital for normal life processes (Rizvi and Saleh, 2018). Actively targeting PLGA nanoparticles are able to circumvent this; Moku et al. (2019) have shown increased drug loading and efficacy against lung cancer by using transactivator of transcription (TAT) peptide ligands to target mesenchymal stem cells while Ganipineni et al. (2019) found that magnetically targeted paclitaxel- and SPIO-loaded PLGA-based nanoparticles effected increased cellular uptake in glioblastoma cells compared to the non-targeted carriers. Another type of nanoparticle targeting is the use of ‘smart’ carriers that are engineered to respond to a stimulus (Kapoor et al., 2015). Recently, a pH dependent aptamer functionalized PLGA nanocarrier system was reported to increase anti-cancer activity of doxorubicin to human lung cancer cells, with reduced toxicity to healthy cells (Saravanakumar et al., 2019) and a superparamagnetic iron oxide encapsulated nanocarrier for docetaxel demonstrated favorable pharmacokinetics and a greater degree of uptake in breast cancer cells (Panda et al., 2019).

#### Immunotherapy

Since Allison and Honjo were awarded the 2018 Nobel prize in Physiology and Medicine “for their discovery of cancer therapy by inhibition of negative immune regulation” (Guo, 2018), the area of nanomedical research into cancer immunotherapy has received substantial attention. This approach involves the use of pharmaceutical agents to activate a patient’s immune system to fight cancers as opposed to traditional chemotherapy which involves directly drugging the cancer cells (Khalil et al., 2016). Chen et al. (2016) have described PLGA nanocarriers equipped with an immunostimulant and photothermal agent, and this formulation showed increased activation of the immune system of BALB mice compared to the free agent. More recently a sustained controlled release PLGA nanosystem was developed to activate the anti-tumor immune response in mice bearing melanoma and colon cancer (Yin, 2019) and a PLGA system containing an immune adjuvant together with an enzyme that increased the efficacy of radiation therapy demonstrated the feasibility of combination immunotherapy and targeted radiotherapy in BALB mice (Chen et al., 2019).

#### Imaging and Diagnostics

Poly lactide-co-glycolide has applications for tumor diagnostics as it is able to deliver imaging agents to cancer cells with specificity and controlled biodistribution. Advances in nanotheranostics, which is the incorporation of imaging and therapeutic agents in one nanocarrier, have shown promise for real time imaging throughout a patient’s treatment course (Chapman et al., 2013). A novel theranostic PLGA nanocarrier with a near infrared imaging agent, further decorated with gold nanoparticles has been synthesized and shown to have increased activity and photodynamic properties in tumor grafted BALB mice (Xi et al., 2018) and a targeted PLGA-based nanobubble was designed with an ultrasound contrast agent, and demonstrated specificity and imaging capabilities to breast cancer in BALB mice (Du et al., 2018). More recently an image guided photothermal PLGA nanocarrier for doxorubicin showed promise for real time photoacoustic imaging in tumor bearing nude mice (Shen et al., 2019) and a near infra-red dye loaded PEGylated PLGA nanocarrier was also able to provide information on the circulation and distribution of the nanoparticles in nude mice (Kumar et al., 2019).

### HIV Treatment

The delivery of anti-retro viral drugs faces many of the general limitations of conventional drug delivery and therefore biomaterials with low toxicity such as PLGA based nanocarriers are being implemented in formulations to treat HIV. Mannosylated PLGA nanoparticle carriers have shown promise for targeted delivery of anti-retro viral drugs to the brain (Patel et al., 2018) and the use of microfluidics technology enabled the novel synthesis of efiravine loaded PLGA nanoparticles (Martins et al., 2019). A recent study reported a PLGA based nanocarrier for the combination of the anti-retro virals griffithsin and dapirivine which showed a long acting treatment profile (Yang et al., 2019) and a separate proof of concept study showed promise for a long acting bictegravir encapsulated PLGA nanocarrier (Mandal et al., 2019).

### Inflammatory Disorders

Many current treatments have proven to be inadequate at treating or alleviating symptoms of inflammation. The specific delivery of anti-inflammatory agents to the target site could potentially increase their therapeutic concentration in the inflamed tissue with reduced side effects (Gendelman et al., 2015), and the use of PLGA is particularly suitable to this application because of its favorable biodegradability and non-immunogenicity (Lamprecht et al., 2001). Davoudi et al. (2018) described a carrier within a carrier system using intestinal organoids to transport 5-ASA encapsulated PLGA nanoparticle to treat inflammatory bowel disease, and Perreira’s research involved the development of a metformin loaded nanoformulation that showed efficacy against periodontal inflammation in diabetic rats (Pereira et al., 2018). Gholizadeh et al. (2018) have formulated a dactolisib-PLGA nanoparticle that showed activity against inflamed endothelial cells and more recently, Yang (2019) group reported the synthesis of a crocetin-loaded nanoparticles that reduced the level of pro-inflammatory cytokines in renal tissue and therefore shows potential for the treatment of diabetes induced nephropathy.

### Other Applications

**FIGURE 12:**
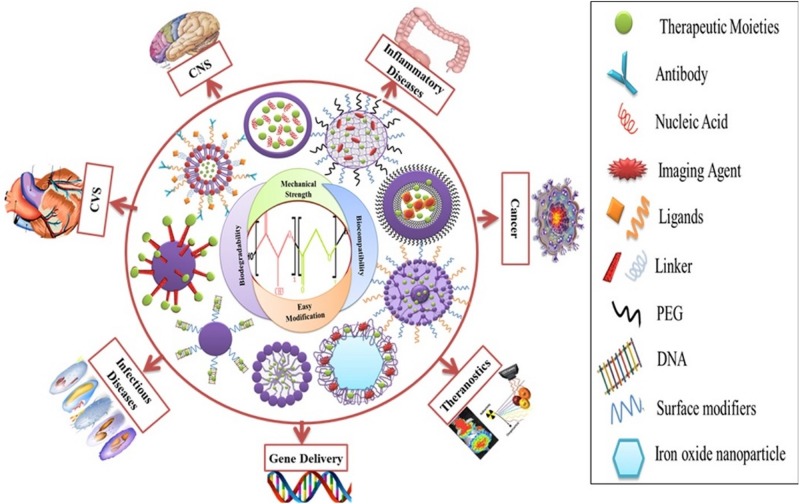
Functionalized PLGA nanocarriers and their medical applications, reproduced from Mir et al. (2017).

Poly lactide-co-glycolide has been adapted to treat conditions in many fields of biomedicine, as shown by Figure 12 (Mir et al., 2017). A hyaluronic acid functionalized PLGA based nanocarrier for methatrexate has been developed for targeted treatment of rheumatoid arthritis (Trujillo-Nolasco et al., 2019), a PLGA nanoparticle with protease inhibitor has shown to overcome gastro-intestinal limitations of oral insulin delivery in rats (Faheem et al., 2019), a PLGA-chitosan based nanocarrier has been synthesized and shown to be selective for human antigen presenting cells (Durán et al., 2019), a potential DNA vaccine delivery system has been designed using a PLGA based nanocarrier (Besumbes et al., 2019), and a Vitamin D encapsulated PLGA based delivery system has recently shown activity against various markers for Alzheimer’s disease in mice (Jeon et al., 2019). Gonzalez-Pizarro et al. (2018) have prepared an optimized fluoromethalone-PLGA nanoparticle that demonstrated increased efficacy in treating ocular inflammation compared to the commercial formula. The use of some of the available methods in PLGA nanocarrier synthesis and their applications are summarized in Table 1.

**TABLE 1:** Properties of some prepared PLGA nanocarriers, their methods of formation and biological targets.

**Type of nanocarrier**	**Route of administration**	**LA:GA in PLGA**	**Mw of PLGA**	**Synthetic Method**	**Particle size range**	**Medical application**	**References**
PLGA nanoparticle embedded in a microsphere	Inhalation	50:50	38.5 K	Single emulsion	∼200 nm	Lung cancer treatment	Guo et al., 2014
PLGA-HDL hybrid nanoparticle	Injectable	50:50	30–60 K	Microfluidics	∼100 nm	Cardiovascular disease treatment	Sanchez-Gaytan et al., 2015
Dye loaded PLGA nanoparticles	Intravenous	50:50	13.5 K	Spray drying	∼500 nm	Photoacoustic imaging	Kohl et al., 2011
Paclitaxel loaded PLGA nanoparticle	Injectable	75:25	694 K	Membrane extrusion	∼300 nm	Malignant melanoma treatment	Liang et al., 2013
Insulin loaded plga nanoparticle	Oral	50:50	10 K	Nano precipitation	∼100 nm	Diabetes treatment	Chopra et al., 2017
Nimodipine loaded PLGA nanoparticle	Intravenous	85:15	–	Modified nano precipitation	∼200 nm	Sustained release for cerebral vasospasm treatment	Mehta et al., 2007
Alendronate loaded PLGA nanoparticle	Intravenous	50:50	40–75 K	Double emulsion	∼300 nm	Restenosis inhibition	Cohen-Sela et al., 2009
Docetaxel loaded PLGA nanoparticles	Injectable	50:50	33 K	PRINT	∼300 nm	Cancer treatment	Enlow et al., 2011

### Inclusion of PLGA Formulations in the Clinic

The biocompatibility, biodegradability and versatility of PLGA has made it suitable for a wide range of clinical applications. PLGA was commercialized in the 1970s as a suture material under the trade name Vicryl^®^ (Kamaly et al., 2016). Other sutures include Dolphin Sutures^®^, and Polysorb^®^ which are both currently approved. PLGA-containing chemotherapeutic formulations approved for clinical use include Lupron Depot^®^, for sustained release of leuprolide, which has application in the management of prostate cancer (Swider et al., 2018), Trelstar^®^, a triptorelin-containing suspension for the treatment of prostate cancer and Zoladex, a goserelin-containing implant used in the treatment of breast and prostate cancer and endometriosis. Formulations approved for other applications include Risperdal^®^ Consta^®^(risperidone), Vivitrol^®^(naltrexone) and Arestin^®^(minocycline) for the treatment of schizophrenia, opioid dependence and periodontal disease respectively (Jain et al., 2016). A promising direction for clinical development is the engineering of PLGA based systems with imaging agents to monitor disease progress and/or relapse patterns using magnetic resonance imaging (MRI). Studies have shown these structures to be non-invasive and cost effective, with excellent safety profiles (Strohbehn et al., 2015). Furthermore, there are a number of PLGA based systems have been used in clinical trials that are ongoing or have been recently concluded (U.S. National Library of Medicine, 2019).

## Computational Modeling

**FIGURE 13:**
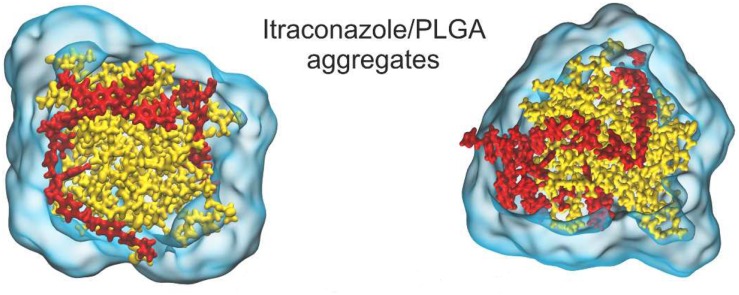
Molecular dynamics (MDs) simulation showing (**A**, left) 12% w/w and (**B**, right) 24% itraconazole loading in a PLGA nanoparticle, adapted with permission from Wilkosz et al. (2018). Copyright (2019) American Chemical Society.

Despite the numerous formulations and methods available for PLGA synthesis, there is a large discrepancy between *in vitro*, *in vivo* and clinical results. One of the reasons for this could be the fact that it is difficult to obtain mechanistic insight into nano-formulation behavior in the various systems by evaluation of results based solely on experimental methods (Huynh et al., 2012). Because of the ubiquity of PLGA across so many biomedical fields of research, there is an abundance of data at our fingertips for computational modeling (*in silico*). There are various levels of detail that can be used in computational simulations. The approach used most widely for nanoparticle drug delivery systems is molecular dynamics (MDs). This technique uses the motion of the molecules in the system to predict its behavior. The parameters it uses are the bonds, bond angles and dihedrals, and here the atoms are treated as point charges. If the degree of detail of atomistic interaction is not required, a coarse grained (CG) model can be used. Here, atoms are grouped into molecular fragments and their behavior in the system is modeled (Frenkel and Smit, 2001). Density functional theory is a model based on electronic density around atoms in the system and measures these interactions within the system of interest (Geerlings et al., 2003). Computational simulations at these levels can give insight into polymer interactions, drug-carrier miscibility, drug loading, drug release, and complex stability (Ramezanpour et al., 2016). Mathematical modeling such as finite element analysis and computational flow dynamics are particular useful when studying polymeric nanoparticle formation (Lince et al., 2011), diffusion and degradation (Kojic et al., 2017). Hence, coupled with experimental methods, they can be powerful tools in the rational design of PLGA nanocarriers for biomedical applications. A study of PLGA binding to curcumin was conducted using MDs with the GROningen MAchine for Chemical Simulations (GROMACS) program, compared to laboratory findings and collated with the experimental results of 10 other PLGA-drug formulations. This study also predicted that PLGA could entrap curcumin with a higher encapsulation efficiency than tripalmitin, a lipid-based carrier, and this prediction was confirmed experimentally (Metwally and Hathout, 2015). A study on the drug release of the anti-cancer drug oxaliplatin in a PLGA matrix was conducted using the Large-scale Atomic/Molecular Massively Parallel Simulator (LAMMPS) program (Lange et al., 2016). A detailed insight into PLGA “patchy particles,” which are particles made up of PLGA and lipid-polymer groups, was obtained from computational fluid dynamics, MDs and coarse grain simulations (Salvador-Morales et al., 2016). A study involving the simulation of PLGA-PEG co-polymer with the hydrophobic drug itraconazole, as shown in Figure 13, provided information about the drug loading limitations of this system (Wilkosz et al., 2018). A MDs simulation of the peptide Melittin showed that it constituted a more stable formulation with PLGA than PLA (Asadzadeh and Moosavi, 2019). The level of these studies as well as the information they provide is summarized in Table 2.

**TABLE 2:** Molecular simulations of PLGA and their significance to nanocarrier drug delivery properties.

**Simulation type**	**Cargo**	**Property modeled**	**Experimental validation**	**Information obtained**	**References**
Density functional theory	Doxorubicin, daunorubicin	Physicochemical properties, binding energy	Qualitative	Carrier-drug affinity, surface modification effects	Rahimi et al., 2012
Molecular dynamics	Curcumin, prednisolone, resveratrol	Binding energy	>85%	Carrier–drug affinity	Metwally and Hathout, 2015
Molecular dynamics	Oxaliplatin	Density Glass transition temperature	>96% >95%	Polymer degradation, drug release	Lange et al., 2016
Molecular dynamics, computational fluid dynamics, coarse grain	None	Shear stress, lipid-PLGA interaction energy	Qualitative	Loading capacity, release kinetics	Salvador-Morales et al., 2016
Finite element analysis	Rhodamine B	Diffusion, drug release	Qualitative	Polymer degradation, drug release	Milosevic et al., 2018
Molecular dynamics	Itraconazole	Binding energy	Qualitiative	Drug loading ability of carrier	Wilkosz et al., 2018
Molecular dynamcs	Melittin	Binding energy, radial distribution function	None	Protein-peptide interactions, encapsulation affinity	Asadzadeh and Moosavi, 2019

Another area of expansion of computational modeling on PLGA nanosystems is be the study of the transport of these nanocarriers in the circulatory system. One of the limitations of the clinical translation of nanosystems in general is the poor correlation between *in vitro* and *in vivo* results. The use of mathematical and computational methods to model the interactions between the drug, carrier, biological transport system and tumor vasculature can be used to gain insight into these complexities (Curtis et al., 2015). Finite element analysis can be employed to model the dynamics of a nanoparticles within a channel, hence simulating transport in a blood vessel while continuum models can also be used to simulate nanoparticles in a vascular network generated by physical input parameters (Liu et al., 2012).

While computational simulations can provide valuable information on the molecular interactions in various PLGA nanocarrier systems, they can be limited by computational cost and time intensive calculations (Ramezanpour et al., 2016). It has been proposed that a minimum reporting standard be instituted where researchers are required to present their results with enough information to make it useful for *in silico* modeling and future work (Faria et al., 2018).

Computational modeling could be immensely useful once it reaches a level where it can be used to select or eliminate certain experimental variables before laboratory research is conducted. Currently, even though simulation time scales are appropriate for the modeling of several nano-systems, the detailed investigation of the formation of nanoparticles by new methods, for example microfluidics, is beyond the abilities of current computational technology. The majority of studies conducted thus far involve the modeling of individual systems; however, more data is needed so that we can move away from specific systems to create profiles to generalize these delivery systems for rational design (Ramezanpour et al., 2016).

Optimizing nanoformulations especially with PLGA polymers which have numerous possible combinations of lactic acid to glycolic acid ratio, molecular weight, endcaps and surface functionalization, could be very time consuming, expensive and in some cases not experimentally feasible. Since computational simulations give a molecular insight to macroscopic properties (Huynh et al., 2012), it could provide a platform to model these initial parameters in order to narrow down the possibilities in a specific study.

## Design Considerations

Several studies have demonstrated the increase in particle size and decrease in drug release rate with increase in molecular weight and lactide:glycolide ratios (Song et al., 2008; Dinarvand et al., 2011). Recently, Lu et al. (2019) designed a 75:25 lactide:glycolide PLGA nanocarrier for the sustained release of paclitaxel. Surface functionalization is a component that needs to factor in when designing nanocarriers. Gu et al. (2008) conducted a study that optimized the *in vitro* release rate of docetaxel in PLGA, and additionally found that they could reduce the size of the nanoparticles from ∼291 to ∼160 nm by shortening the length of the PEG chains that were used for surface functionalization, while Bertrand et al. (2017) found that up to a point, increasing the density of PEG surface functionalization increased the blood circulation time of their nanoformulations. Gu’s group also investigated an optimum targeting ligand density in order to provide maximum targeting ability without inhibiting the shielding effect of the PEG corona (Gu et al., 2008), while Lu’s group found that increasing the density of the chitosan coating in their formulation increased the particle size from ∼133 to ∼173 nm (Lu et al., 2019).

Since the choice of fabrication methods and processes can determine the physicochemical characteristics of the resulting system, it is important to select an approach that is associated with the desired nanoparticle properties for the system of interest. For example Kim S.R. et al. (2019) reported that even though the preparation of their entacavir-loaded system by spray drying produced larger particle size diameters compared to emulsion techniques, the spray dried system showed a much more favorable drug loading and release profiles and hence was the better performing delivery system. Krishnamoorthy described a multi-criteria decision making approach to the synthesis of polymeric nanoparticles which concluded that nanoprecipitation would be the best suited preparation method for a campthothecin-loaded system (Krishnamoorthy and Mahalingam, 2015), and an adapted approach could be implemented in the selection of synthetic methods for specific PLGA-based systems.

## Disadvantages of PLGA as a Nanocarrier

Even though the versatility of PLGA makes it an attractive option as a nanocarrier, it does present several challenges in nanomedicine. PLGA co-polymers are usually readily commercially available, but to obtain it in a high purity, and the specificity required for different molecular weights, lactic/glycolic acid ratios and end capped options can make it very costly (Danhier et al., 2012). Many formulations show poor drug loading and therefore would require large doses in order to achieve therapeutic concentrations of cargo at the target site. Furthermore, these systems often exhibit burst release kinetics, which would result in off target *in vivo* delivery. The degradation rate of PLGA is often unpredictable and the acidic degradation products have shown to affect the activity of the encapsulated drug (Sharma et al., 2016), and despite its biodegradability, reports have shown that the use of PLGA in medical devices may produce localized reaction at the site of delivery (Makadia and Siegel, 2011). Even though targeted PLGA based carriers theoretically have more efficient site specific delivery properties, the targeting moieties in these nanosystems can induce additional immunogenocity (Danhier et al., 2010). There are various *in vivo* physiological barriers and up- and down-regulation of cell surface receptors and other targets can also decrease the efficacy of the targeting agents in these systems. The adaptability of PLGA as a polymer for its specific application has resulted in it being used in many delivery systems and therefore it is difficult to make comprehensive predictions at this stage about its general behavior and toxicity (Sharma et al., 2016).

## Conclusion and Future Work

Even though PLGA is a polymer with many desirable features, there are various areas in which research can be conducted to improve the viability of PLGA based nanocarriers for clinical translation. Since PLGA has been developed in drug delivery systems for such a wide berth of applications, it should be precisely designed in terms of cargo suitability, particle size, drug entrapment and degradation kinetics, for its specific target. This would determine the choice of starting materials and in some cases, the method of preparation and would therefore remove some of the uncertainty present in several trial-and-error attempts in previous drug delivery systems. The innovative fabrication techniques mentioned above could also be attempted to increase control over homogeneity of the products. The use of *in silico* modeling for PLGA nanoparticles as an element of experimental design and could have tremendous implications for the future of nanoparticle design.

## Author Contributions

DE, PK, YC, and VP from designing the framework and main content of the manuscript, revisions to optimize the manuscript, approved the final submission, and the manuscript was accomplished with contributions.

## Conflict of Interest

The authors declare that the research was conducted in the absence of any commercial or financial relationships that could be construed as a potential conflict of interest.
